# Supplementary material for: Acoustic Anomalies and the Critical Slowing-Down Behavior of MAPbCl3 Single Crystals Studied by Brillouin Light Scattering
Source: Materials (Basel). 2022 May 21;15(10):3692. doi: 10.3390/ma15103692 (PMC9145713; doi:10.3390/ma15103692)

Supplementary Materials

Acoustic Anomalies and the Critical Slowing-Down Behavior of $MAPbCl_3$ Single Crystals Studied by Brillouin Light Scattering

Jeong Woo Lee, Furqanul Hassan Naqvi, Jae-Hyeon Ko*

School of Nano Convergence Technology, Nano Convergence Technology Center, Hallym University, Chuncheon, Gangwondo 24252, Republic of Korea

Tae Heon Kim and Chang Won Ahn

Department of Physics and Energy Harvest-Storage Research Center (EHSRC), University of Ulsan, Ulsan, 44610, Republic of Korea

* Correspondence: email: hwangko@hallym.ac.kr

Figure S1. comparison of (a) the Brillouin frequency shift and (b) the FWHM with the real part of the dielectric permittivity reported in Ref. 31.

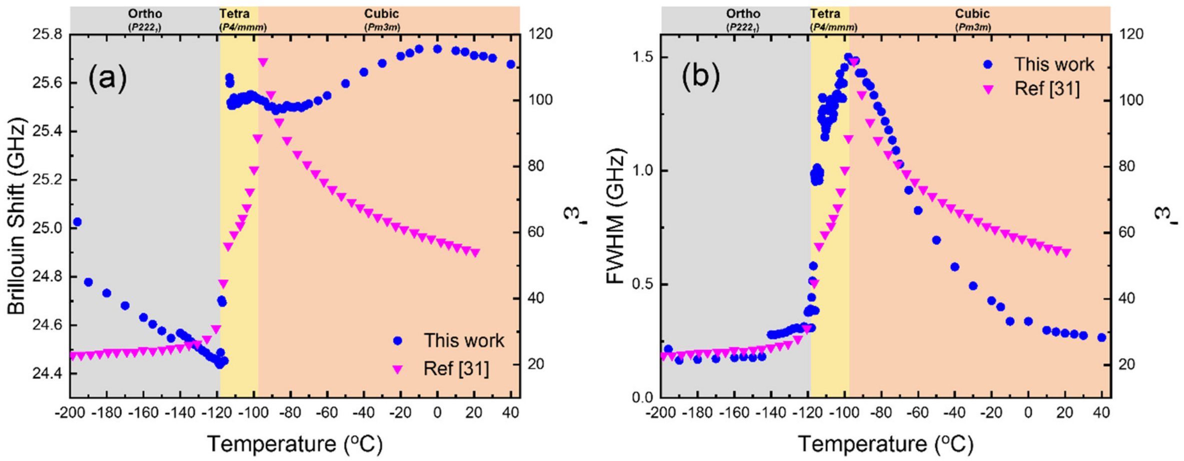

Figure S2. Temperature dependences of (a) the mode frequency and (b) the FWHM of the TA mode propagating along the [100] direction measured upon cooling and heating.

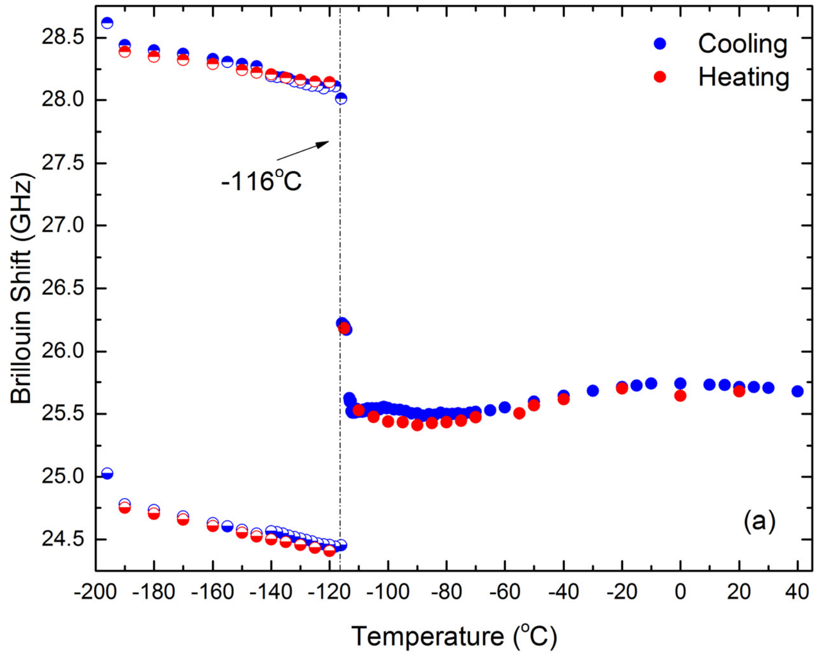

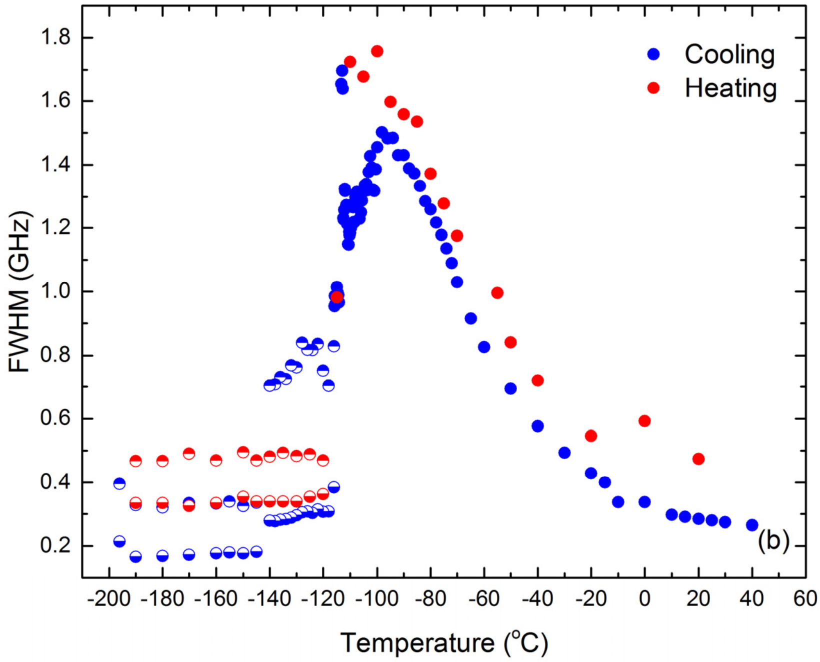

Figure S3. Temperature dependences of (a) the mode frequency and (b) the FWHM of the TA mode propagating along the [100] direction measured upon cooling and heating.

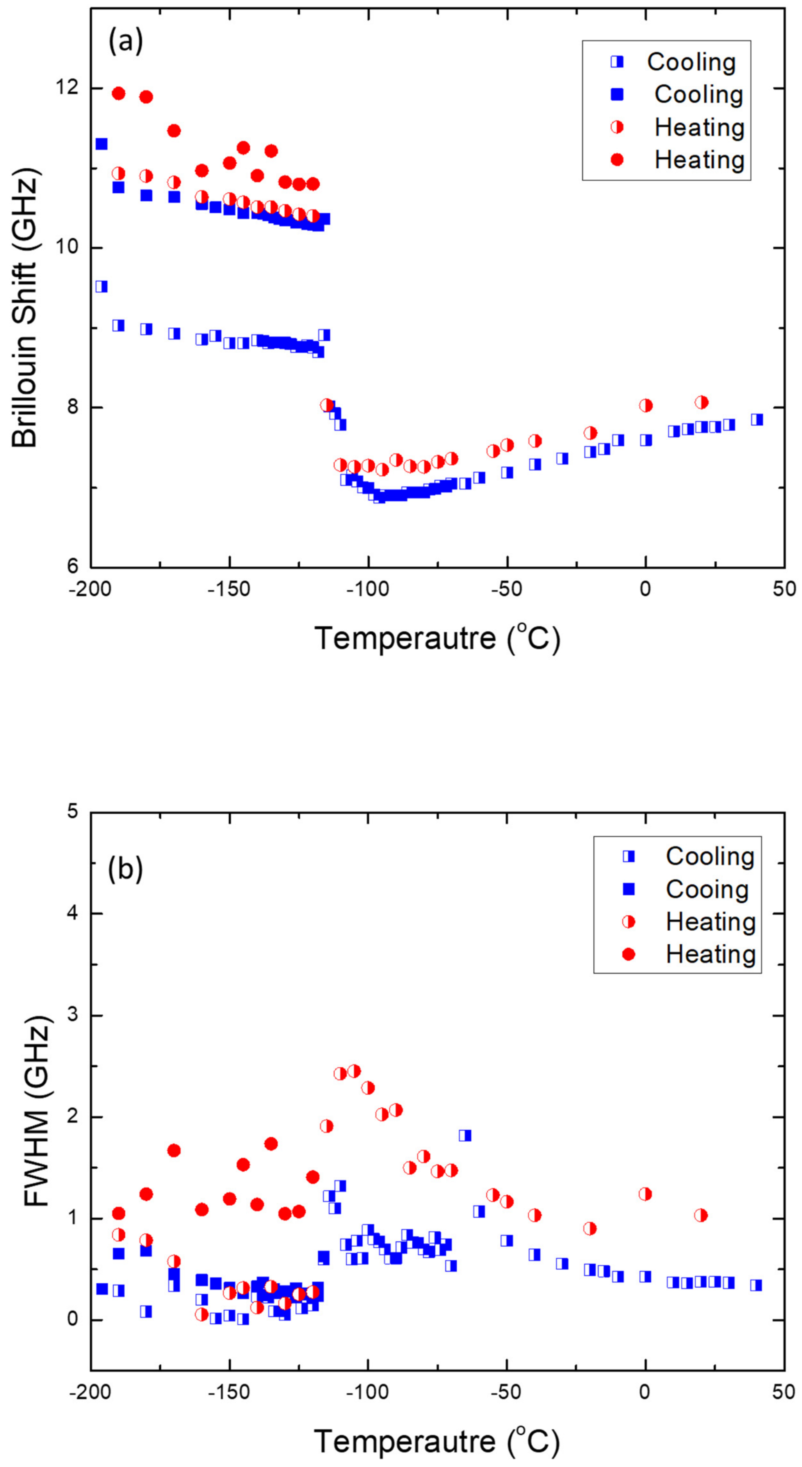

Supplement: Supplementary file 1 [file materials-15-03692-s001.zip › materials-1725295-supplementary.pdf]
